# Prospect of Muscle-Building Supplement HMB in Alzheimer’s Disease

**DOI:** 10.33140/jcei.11.03.01

**Published:** 2026-07-13

**Authors:** Abhiram Uppalapati, Ahiri Vishnubhotla, Kalipada Pahan

**Affiliations:** 1 Division of Research and Development, Jesse Brown Veterans Affairs Medical Center, Chicago, IL; 2 Department of Neurological Sciences, Rush University Medical Center, Chicago, IL

**Keywords:** Alzheimer’s Disease, HMB, Amyloid Plaque, Hippocampal Plasticity, CREB, PPARα, ADAM10, TFEB, Autophagy

## Abstract

Alzheimer’s disease (AD) is the most common progressive and irreversible neurodegenerative disorder in humans that affects memory, thinking and behavior. Impairment in synaptic plasticity is one of the hallmarks in AD, with most of the impairment occurring in the hippocampal region, a key part of the brain for memory and learning. Therefore, the upregulation of hippocampal plasticity is critical to remediate the progression of AD and preserve memory formation and cognitive functions. Recent studies have described β-hydroxy-β-methylbutyrate (HMB), a body building supplement commonly used by athletes, as a candidate molecule for improving hippocampal plasticity. Clinically, AD is characterized by the abnormal accumulation of beta amyloid (Aβ) plaques, coupled with intracellular aggregates of hyperphosphorylated tau protein. In addition to enhancing hippocampal plasticity, HMB has been also demonstrated to lower amyloid plaques in a mouse model of AD. Although liver is rich in peroxisome proliferator-activated receptor alpha (PPARα), recent findings have established the presence of PPARα in hippocampus and other parts of the brain. Interestingly, HMB has been shown to utilize PPARα for lowering plaques and increasing hippocampal plasticity. Here, we discuss these newly described features of HMB with possible implications for the use of HMB supplement in patients with dementia and AD.

## 1. Introduction

Alzheimer’s disease (AD) is attributed to being a common cause of dementia, comprising 60–70% of cases worldwide [1]. Despite extensive research, outcome altering treatments remain limited, and current therapies provide only somewhat of relief from symptoms. Thus far, attention about AD has focused on early pathological processes and resulting pathophysiology such as synaptic dysfunction, impaired neuroplasticity, and significant neuronal loss [2–5]. The hippocampus is a pertinent brain region in memory encoding and consolidation, and this makes it one of the earliest brain regions affected in AD. Thus, retardations in hippocampal plasticity correlate with cognitive decline, even prior to plaque depositions [6,7]. Consequently, therapeutic strategies that preserve or restore hippocampal plasticity are emphasized and are of considerable interest.

Nutritional and metabolic strategies are gaining popularity as potential ways to improve brain health. Specifically, β-Hydroxy-β-methylbutyrate (HMB) is a leucine-based metabolite known for its anabolic and anti-catabolic effects in skeletal muscle [8]. However, recent findings attribute HMB’s significance in relation to the central nervous system [9–12]. HMB can regulate transcriptional pathways, and this is crucial to neuronal survival and synaptic restructuring. Notably, Paidi et al. have demonstrated that HMB activates PPARα signaling in the hippocampus, resulting in enhanced neuroplasticity, better cognitive function, and decreased amyloid plaque pathology in a mouse model of AD [11]. These findings indicate that HMB may be a safe and accessible approach to addressing early synaptic dysfunction in AD.

## 2. Alzheimer’s Disease

Alzheimer’s disease (AD) is a neurodegenerative disorder determined by progressive deficits in memory and executive function. AD is pathologically characterized by the aggregation of extracellular amyloid-β (Aβ) plaques, intracellular neurofibrillary tangles comprised of hyperphosphorylated tau, synaptic diminutions, and neuroinflammation [13–19]. The amyloid cascade hypothesis theorizes that abnormal Aβ accumulation initiates downstream events such as synaptic dysfunction, changes in tau pathology, and neuronal apoptosis [13–15,20–22]. However, recent evidence shows that soluble Aβ oligomers can be harmful to synapses and can impede plasticity mechanisms crucial for learning and memory [23]. Metabolic lapses, oxidative stress, mitochondrial impairment, and neuroinflammation can impact the progression of AD [17,19,24–26]. In conjunction with each other, these processes worsen neuronal vulnerability, and these effects pose a risk in energy intensive regions like the hippocampus [27]. Moreover, synaptic plasticity deficits occur early and correlate more strongly with cognitive decline than neuronal lapses or plaque accumulation [28–30].

Popular pharmacological treatments such as cholinesterase inhibitors and NMDA receptor antagonists are limited solutions and do not substantially reduce disease progression. Recently approved anti-amyloid therapies reduce plaque accumulation, but the crux is that they also pose safety concerns and lack of clarity when it comes to long-term cognitive benefits. These limitations emphasize the need for strategies that target synaptic and metabolic health and neuroplasticity. Interventions that support hippocampal structure and function, particularly during early AD, may slow cognitive decline, thus improving quality of life. And in this context, substances such as HMB that impact transcriptional pathways relevant to neuroplasticity warrant attention.

## 3. Hippocampal plasticity

The hippocampus, located deep in the brain within the medial part of the temporal lobe, is known for its malleability (known more commonly as plasticity) where it can adapt both structural and functional properties in response to various environmental and physiological factors [15,31]. Long-term potentiation (LTP) is a persistent increase in synaptic strength typically induced by high-frequency stimulation of various chemical synapses [5,32,33]. LTP in hippocampal circuits (for example at CA1 synapses) is considered a prominent cellular model of learning and memory [34]. Conversely, long-term depression (LTD) is a lasting decrease in synapses with low-frequency stimulation [35]. Studies reveal that hippocampal plasticity heavily depends on the coordinated regulation of PSD-95 (which stabilizes NMDA/AMPA receptors at the postsynaptic site), SNAP-25 (which mediates presynaptic vesicle fusion and neurotransmitter release), and the balanced cohesion of these glutamate receptor subunits, all of which jointly govern synaptic strength and efficacy [36,37]. It has been shown that beyond Ca2+ entry through NMDA and voltage-gated channels, synaptic activation could mobilize additional Ca2+ from intracellular stores via metabotropic glutamate receptor (mGluR)-activated IP3 receptors and calcium-induced Ca2+ release through ryanodine receptors: important in driving LTP and LTD [38]. This is accompanied by the activation of kinases (i.e. CaMKII), changes in gene expression, and growth of new synaptic connections [39]. Plasticity is regulated by various molecular players. For example, brain-derived neurotrophic factor (BDNF) supports spine maintenance and growth, while intracellular enzymes such as glycogen synthase kinase-3beta (GSK3-beta) and CREB (cAMP response element-binding protein) modulate the plasticity processes [4,30,40–42]. Disruption of these plastic mechanisms is implicated in cognitive disorders, especially AD.

## 4. Role of hippocampal plasticity in AD

In AD, the hippocampus is among the earliest and most profoundly affected regions [36,43]. Numerous sources show that the pathological hallmarks of AD (e.g. extracellular Aβ plaques and intracellular neurofibrillary tangles composed of hyperphosphorylated tau) exhibit a detrimental effect on hippocampal plasticity [36,43]. When Aβ oligomers form, they disrupt the balance between excitatory and inhibitory signaling by interfering with post-synaptic receptor function (ex. NMDA/AMPA), thereby impairing synaptic plasticity [37,38]. On the other hand, with the increase in Aβ plaques, microglia respond with a pro-inflammatory phenotype, releasing cytokines that negatively affect synaptic stability and reduce overall neurogenic capacity of the dentate gyrus [31,40].

This apparent breakdown of synaptic and cellular plasticity converges with more downstream alterations in signaling pathways. BDNF is compromised, leading to limited neuronal survival, synaptic maintenance, and dendritic spine growth [36]. Dysregulation of GSK-3β (seen with tau phosphorylation) exacerbates cytoskeletal damage, while attenuating CaMKII and CREB mediated gene expression required for long term synaptic remodeling. Fewer new granule neurons successfully mature and integrate, and existing synaptic circuits suffer progressive atrophy [31]. The hippocampus’ intrinsic ability to reorganize in response to stimuli steadily diminishes, correlating clinically with the well-known deficits in episodic memory and spatial navigation seen in AD patients. Over time, repeated insults (from chronic inflammation to oxidative stress) eventually erode the hippocampal plastic response [40]. Although some remnants of neurogenic capacity may remain, it typically falls below the threshold needed to compensate for ongoing neuronal loss and synaptic failure [40]. Thus, the vicious cycle of impaired plasticity contributes to the accelerating cognitive decline that characterizes AD.

## 5. Muscle-building supplement HMB

Leucine is a powerful activator of muscle growth and plays a key role in building new muscle tissue and beta-hydroxy-beta-methylbutyrate (HMB) is a metabolite of leucine [44]. HMB is widely found in many retail stores as a body-building supplement. Body builders use HMB to increase muscle size and strength, and to improve exercise performance because HMB acts as an anticatabolic agent to minimize protein breakdown to cells [45]. HMB is known to support bone density, reduce abdominal obesity, and improve cognitive function-important in the older population [46]. HMB appears to be most beneficial to untrained individuals or during periods of high training stress, where it can significantly reduce muscle breakdown [47]. The supplement has also been proved to enhance sarcolemma integrity, inhibit protein degradation, decrease cell apoptosis, and increase the mTOR pathway [48]. Various HMB studies have shown that this supplement improves the proliferation of muscle cells and has an effect on muscle strength [49]. In a 12-week randomized, double-blind, placebo-controlled crossover study among 42 highly trained combat-sports athletes, HMB supplementation significantly increases fat-free mass while decreasing fat mass compared with placebo [50]. With regard to this, HMB is widely considered a safe and useable supplement in humans. Studies consistently report minimal or no adverse effects even with prolonged use, reinforcing its status as a safe supplement.

## 6. Stimulation of hippocampal plasticity by HMB

**Figure 1:**
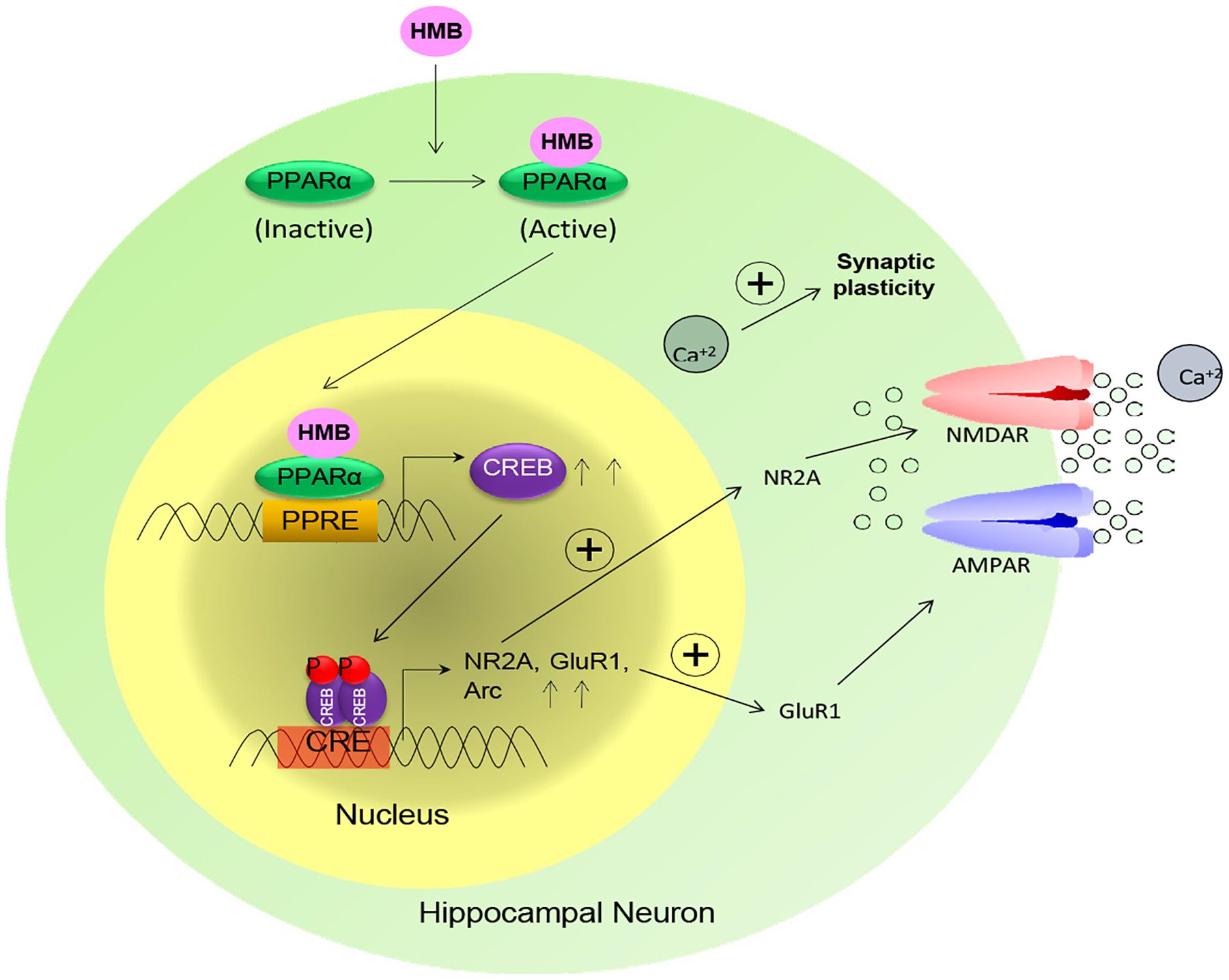
Upregulation of hippocampal plasticity by HMB. HMB activates PPARα, which binds to the promoter of CREB gene in the nucleus. The CREB is a transcription factor that turns on the transcription of different plasticity-related molecules including NR2A and GluR1, resulting in the upregulation of calcium influx and improvement in synaptic plasticity.

Recent evidence shows that HMB has great neuroprotective effects in the hippocampus with synaptic protein upregulation, enhancing dendritic spine density and reducing amyloid pathology in mouse models of AD [11]. Despite widespread interest on HMB, nothing was known about a receptor HMB. Structural analyses together with thermal shift assay, time-resolved fluorescence resonance energy transfer assay, and site-directed mutagenesis have pinpointed tyrosine 314 (Y314) within the ligand-binding domain of peroxisome proliferator-activated receptor alpha (PPARα) as crucial for HMB binding [11]. Upon binding, HMB activates PPARα-dependent transcriptional programs that in turn increase the expression of CREB, the master regulator of memory and learning (Figure 1). Activation of CREB then leads to the expression of synaptic plasticity related proteins such as NR2A and GluR1, leading to rhythmic fluctuations in intracellular Ca2+ concentration, key signal for synaptic plasticity (Figure 1). Moreover, HMB-induced CREB may also lead to the transcription of other plasticity-related molecules such as, PSD-95, SNAP-25, BDNF, etc. in hippocampal neurons [11].

In primary hippocampal cultures, HMB is seen to increase dendritic spine density and spine size, key indicators of morphological plasticity. These findings align with well-established models wherein robust dendritic spine architecture underpins effective synaptic connectivity and information processing. HMB restores synaptic strength in hippocampal slices from transgenic 5XFAD mice, offsetting known deficits in NMDA/AMPA receptor mediated Ca2+ signaling in AD [11]. These benefits are absent in PPARα-deficient models, confirming that HMB-PPARα interaction is needed for its hippocampal actions [11]. By coupling structural augmentation of synapses to transcriptional programs that preserve neuronal health, HMB may emerge as a possible candidate for preventative interventions in regard to the hippocampal dysfunction in AD.

## 7. Protection of memory and learning by HMB in an animal model of AD

In the 5XFAD transgenic mouse model of AD, oral HMB treatment significantly improves hippocampal-based learning and memory [11]. Behavioral assessments show improved performance in spatial learning and memory tasks in 5XFAD mice compared with untreated mice. HMB treatment increases the level of PPARα in the hippocampus of 5XFAD mice [11]. It has been also shown that HMB-mediated cognitive improvements were due to the activation of PPARα as knock down of PPARα dissipates HMB’s efficacy of improving memory and learning [11]. This finding establishes the role of PPARα signaling in HMB’s ability to effectively transcriptionally regulate and influence cognitive benefits. HMB improves memory performance without producing stimulatory or anxiety reductive effects, eliminating the possibility of attributing performance to behavioral factors rather than actual improvement in hippocampal function. These results are in alignment with the concept that restoring synaptic plasticity has significant cognitive benefits even in the presence of underlying AD pathology.

## 8. Reduction of amyloid plaque pathology by HMB in an animal model of AD

Oral HMB treatment is also seen to reduce Aβ plaques in AD models. In 5XFAD mice, which exhibit a rapid buildup of both Aβ42 and Aβ40 isoforms, oral supplementation of HMB substantially reduces plaque deposition throughout the hippocampus and cortex [11]. Histological analyses including 6E10 immunostaining and thioflavin-S labeling reveal fewer and smaller amyloid plaques in HMB-treated mice as compared to control animals [11]. Since plaque accumulation is an early and central driver to AD pathology, intervention with HMB may lower amyloid burden and minimize downstream neuroinflammation, ultimately providing long-term neuroprotective benefits.

**Figure 2:**
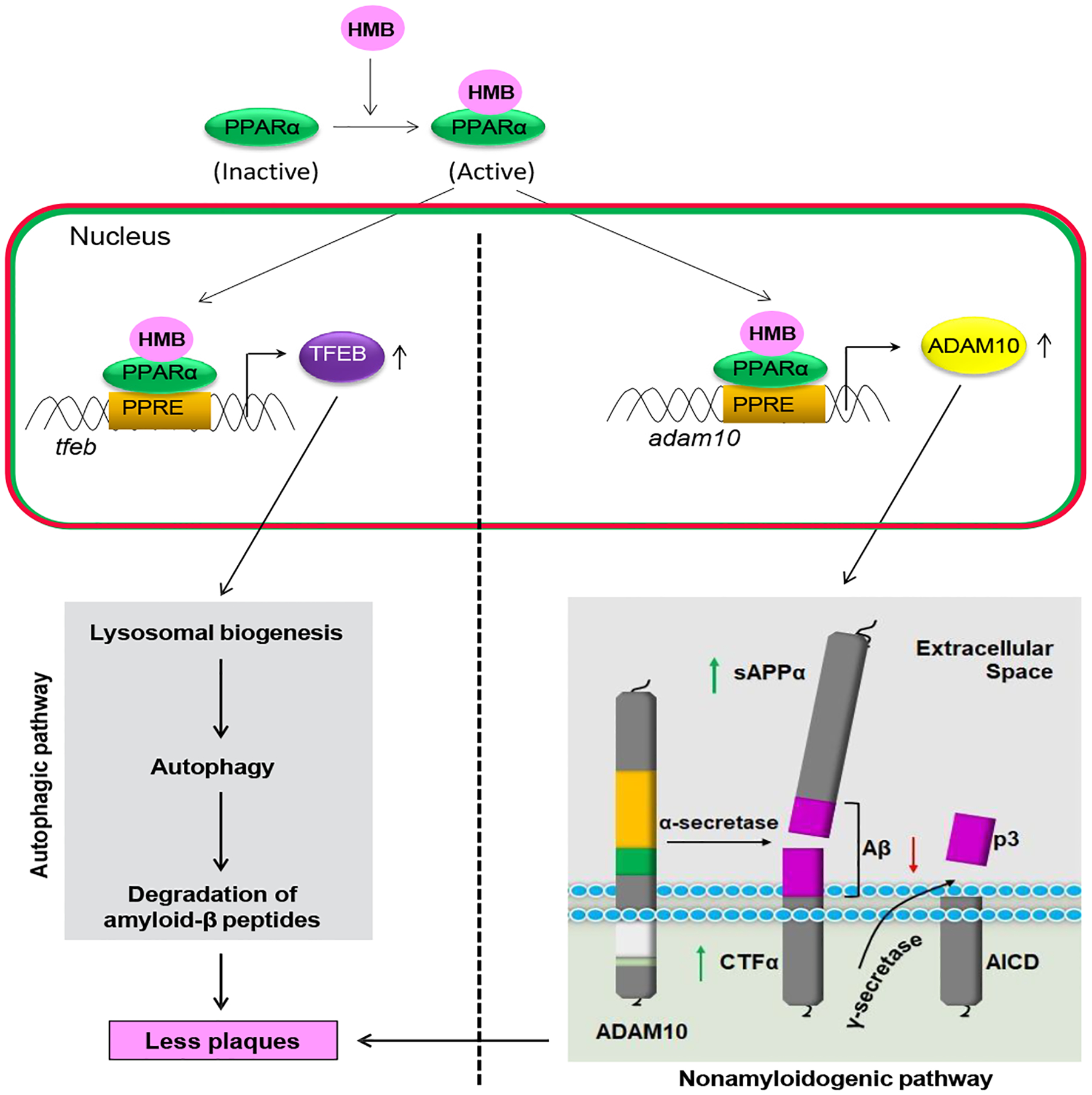
Possible mechanisms behind HMB-mediated reduction of plaque load. While non-amyloidogenic cleavage of APP is driven by ADAM10, the transcription factor EB (TFEB) leads to lysosomal biogenesis and autophagy. HMB activates PPARα acting as a ligand. Then PPARα causes transcriptional upregulation of ADAM10 and TFEB, ultimately leading to the activation of both non-amyloidogenic pathway and autophagic clearance of Aβ. APP, amyloid precursor protein; AICD, APP intracellular domain; ADAM10, a disintegrin and metalloproteinase; CTFα, C-terminal fragment alpha; PPRE, peroxisome proliferator response element; sAPPα, soluble APP alpha.

Reduction in plaques from the brain is regulated by both plaque formation as well as degradation of existing plaques (Figure 2). While the upregulation of transcription factor EB (TFEB)-driven lysosome-autophagy pathway results in the degradation of amyloid plaques, the increase in α-secretase “a disintegrin and metalloproteinase” 10 (ADAM10)-driven nonamyloidogenic pathway involving soluble amyloid precursor protein alpha (sAPPα) hinders the formation of amyloid plaques in neurons [51–54] (Figure 2). Although it has not been checked, HMB may upregulate both TFEB and ADAM10. Being a lipid-lowering transcription factor, PPARα is known to reduce the level of free fatty acids and triglycerides via upregulation of peroxisomal β-oxidation pathway [55–57]. Therefore, liver is rich in PPARα. However, we have established the existence of PPARα in different regions of the brain including the hippocampus [58–60]. Among other newly-described functions in the brain, this PPARα plays a key role in controlling both plaque formation and plaque degradation [52,61]. Interestingly, promoter of both TFEB and ADAM10 genes harbor peroxisome proliferator responsive element (PPRE) (Figure 2). As a result, while in one hand, activation of PPARα stimulates the nonamyloidogenic pathway via transcriptional upregulation of ADAM10, activated PPARα has also been shown to increase lysosomal biogenesis and autophagy via transcriptional stimulation of TFEB [52,53,61]. Accordingly, different molecules and procedures capable of activating PPARα readily increases the level of ADAM10 and TFEB [14, 62–66]. Since HMB binds to tyrosine 314 residue of PPARα ligand-binding domain for the activation of PPARα, HMB should be able to upregulate both ADAM10 and TFEB (Figure 2).

## 9. Therapeutic Prospect of HMB in AD

HMB’s oral bioavailability and low toxicity position this molecule as a promising candidate for long-term administration in older adults, a key demographic that is most susceptible to AD [44,67]. HMB could be utilized alongside existing standard of care treatments such as cholinesterase inhibitors or NMDA receptor antagonists, providing a multifaceted approach. HMB’s ability to modulate multiple pathways like oxidative stress, neuroinflammation, and dysregulated protection aggregation may offer much broader neuroprotection than single target interventions [68]. Future research should clarify optimal dosing regimens, longitudinal safety in diverse AD populations, and the degree of functional synergy with other neuroprotective agents.

More importantly, therapeutic efficacy relies on intact and modifiable PPARα signaling [11]. Therefore, identifying patient subgroups with favorable PPARα activity may enhance precision in clinical applications. HMB’s unique combination of PPARα-mediated neuroprotection, anti-amyloid properties, and minimal side effects underlies its strong therapeutic potential for AD. Further clinical trials are essential to translate promising preclinical findings into viable patient interventions.

## 10. Conclusion

AD is defined by early synaptic impairment and dysfunctional hippocampal plasticity, which serve as indicators of cognitive decline. AD remains an urgent clinical challenge due to its complex pathophysiology and the absence of curative treatments. Disease modification is seen as a viable strategy, and targeting the processes responsible for disease progressions seems to be a promising avenue. β-Hydroxy-β-methylbutyrate, a muscle-building supplement, has been of interest because of its ability to influence hippocampal plasticity through activation of PPARα. Preclinical evidence indicates that HMB improves neuronal morphology, improves learning and memory, and mitigates amyloid accumulation in an animal model of AD. Moreover, HMB’s favorable safety profile and oral bioavailability makes it a promising therapeutic intervention for long-term use in older populations. Since HMB modulates multiple pathways, its incorporation alongside conventional therapies may yield synergistic benefits. The requirement of intact PPARα signaling underlies the importance of patient stratification, as well as further clinical trials to establish optional dosing regimens and assess enduring outcomes. HMB’s dual capacity to preserve hippocampal function and to counteract fundamental AD pathologies positions it as a valuable therapeutic avenue worthy of continued investigation and potential translation into clinical practice for preserving brain function and slowing down age-related neurodegeneration.
